# Cognitive and physiological impacts of psychotherapy incorporating human-equine interactions during substance withdrawal: A scoping review

**DOI:** 10.1016/j.eqre.2025.100022

**Published:** 2025-03-03

**Authors:** M.M. Friend, M.C. Nicodemus, C.A. Cavinder, C.O. Lemley, P. Prince, K. Holtcamp, R. Elam

**Affiliations:** a Huck Institutes of Life Sciences, The Pennsylvania State University, 101 Huck Life Sciences Building, University Park, PA 16802, USA; b Department of Animal and Dairy Sciences, Mississippi State University, PO Box 9815, MS 39762, USA; c Office of Psychological Services, College of Veterinary Medicine, Mississippi State University, PO Box 6100, MS 39762, USA; d Department of Psychology, Mississippi State University, PO Box 6161, MS 39762, USA

**Keywords:** Addiction, Substance withdrawal, Psychotherapy, Human-equine interaction

## Abstract

The use of psychotherapy incorporating equine interaction (PIE) is becoming widely utilized for the treatment of a range of conditions; however, evidence supporting the efficacy of this treatment is highly variable, inconsistently defined, and largely anecdotal. Further, few studies have investigated its use in the withdrawing substance use disorder (SUD) population. This scoping review investigates the physiological and cognitive implications of withdrawal associated with SUD in conjunction with the effects of psychotherapy utilizing equine interaction on these parameters. This review was developed following the guidelines of Preferred Reporting Items for Systematic Reviews and Meta-Analyses (PRISMA) scoping review protocol. The search utilized Google Scholar, PubMed, Medline, and PsychInfo databases to collect literature. Of the 556 articles within the initial review, 153 papers were reviewed in full and 122 met inclusion criteria. The information from this literature indicated addiction and withdrawal chronically elevate stress responses such as cortisol. This elevation has a negative impact on cognitive functions integral to recovery. Psychotherapy incorporating equine interaction has been cited to mediate these symptoms through potential physiological coupling, thus, supporting conclusions from literature that this treatment improves recovery rates. The literature revealed enhancements in therapeutic alliances, patient comfort, confidence, mental health, emotional stability, and communication skills associated with this therapeutic intervention. As such, the literature included in this review supports the potential for the use of psychotherapy incorporating equine interaction in SUD treatment, particularly during the period of withdrawal. However, there is a lack of literature investigating physiological impacts of PIE, especially within SUD treatment.

## 1. Introduction

### 1.1. Rationale

The United States entered a drug epidemic in the 1990s as waves of prescription drugs washed over the population. This triggered a dilemma spanning decades and claiming over half a million lives [1]. Half the American population has reported using illicit drugs at least once, and 25.4 % of these individuals have been diagnosed with substance use disorder (SUD). A consequence of SUD prevalence is the tragedy of overdose, a fate that has claimed over 700 thousand American lives [2]. These numbers were compounded with the onset of the COVID-19 pandemic, as it revitalized addiction throughout the country. A reported 13 % of Americans either initiated or increased substance use during the pandemic, leading to an 18 % rise in overdoses [3], thus, increasing the urgency today for an effective intervention.

### 1.2. Treatment options for substance abuse

Effective treatment options are essential for combatting SUD, but these treatments typically require patient retainment within residential treatment programs over 90 days. This time commitment is necessary for the instillment of essential skills needed to promote sober habits [4]. Addiction relapse occurs in 40–60 % of cases and is directly correlated with treatment facility dropout rates [5]. Substance detoxification is a necessary first step to sobriety, though it is noted as one of the most difficult hurdles to recovery. The difficulty of this process is responsible for a significant percentage of treatment dropouts [6]. Thus, treatment plans designed to ease withdrawal symptoms, and in turn, increase retention rates are necessary elements in the fight against the substance abuse epidemic gripping the country.

A characteristic of effective treatment is the development of positive routines and habits to replace the habitual compulsions of addiction. Failure to overcome the Pavlovian conditioning of addiction is predictive of relapse [7]. In fact, the evaluation of addiction severity published by The American Psychiatric Association is highly reflective of a lack of control and susceptibility to compulsive behavior [8]. Heightened cortisol may play a causative role in the development and retention of habitual behavior in addiction and early recovery [9]. Sufferers of SUD see a decline in facets of executive memory and cognition, potentially further inhibiting their ability to override substance-seeking behaviors [10,11]. According to Baddeley [12], executive memory refers to functions such as planning, working memory (i.e., the ability to intake and process new information), inhibition, concept formation, and set-shifting. Cognition as defined by Miller et al. [13] is defined as the ability to take in and process information throughout several processes and to different degrees. The decline in these abilities is due to the pervasion of the habit system in the brain, and this pervasion may be attributed to heightened cortisol concentrations present during addiction and withdrawal [14,15].

It is widely known that cortisol has an inverted U-shape relationship with both cognition and memory when evaluated separately, thus, exceedingly low and high concentrations circulating in the body are detrimental to these functions [15-17]. Research has also largely agreed interventions blocking cortisol receptors responsible for the hormone’s effects improve cognitive performance [18-20]. Detoxification and maintained sobriety lead to eventual reduced cortisol concentrations and recovery of cognitive function; however, focus on expediting cognitive recovery in therapeutic intervention may improve program retention and encourage prolonged sobriety. Further understanding of the physiological components contributing to cognitive performance may help identify how to develop this treatment focus.

Psychotherapy incorporating equine interaction (PIE) is rapidly growing as an alternative therapeutic approach for mental health disorders including youth with attention deficit hyperactivity disorder, veterans with post-traumatic stress disorder (PTSD), and more recently, addicts seeking treatment [21-23]. This type of therapy capitalizes on the calming effect horses have on humans to facilitate therapeutic interventions and teach adaptive behaviors for functional life skills such as self-care and grooming, danger avoidance, and relationship maintenance. During this interaction, a mental health professional conducts a therapy session using the horse as a complementary therapeutic mechanism. While research is available promoting the positive impacts of this form of therapy can have on SUD patients [21,24], there is little research investigating therapeutic programs utilizing equine interaction that are specifically targeting substance withdrawal. Nevertheless, research within the last decade has revealed this method of therapy could increase patient retention and program completion [21], but again, these studies are limited.

The predominant challenge of SUD rehabilitation is the detriment of drug use and withdrawal-related stress to cognitive function, as it in-hibits the development of new coping mechanisms that may require abilities such as decision-making or goal-directed behavior. A great deal of literature surrounding this topic has recognized a significant relationship between cognitive deficits and cortisol concentrations. Thus, mediation of physiological stress responses to substance abuse may improve cognitive performance as well as the severity of withdrawal symptoms to increase retention rates and sobriety. Research concerning physiological synchronization during human-animal interactions gives insight to a potential therapeutic tool for combatting these stress responses hindering SUD recovery [25-29]. The impact of human-animal interactions and this associated physiological coupling effect could potentially mitigate the exacerbation of these stress responses during withdrawal. Studies evaluating the physiological benefits of stress-related cortisol and heart rate coupling during equine interaction in conjunction with literature investigating the impact of these measures on withdrawal symptoms may point to this therapeutic invention as a method of alleviating stress responses and improving symptom severity during withdrawal.

### 1.3. Review aims

The objective of this review is to draw connections between fields of research to suggest the aptitude of PIE for the treatment of SUD that targets the withdrawal process. This scoping review aims to provide information concerning the following: 1) physiological benefits of this form of therapy, 2) correlating cognitive and emotional impairments within substance addiction withdrawal and treatment, and 3) current research investigating the use of human-equine interaction within SUD treatment. Current literature on the implementation of equines as a treatment tool for mental health professionals during substance withdrawal will be evaluated, and future research considering overlapping themes will be proposed.

### 1.4. Methods and design

The purpose of this paper is to provide evidence spanning multiple research fields to suggest PIE as a SUD treatment option, particularly through the process of withdrawal. Research concerning the impact of withdrawal on cortisol, the effect of cortisol on cognition, and existing research on physiological impacts and synchronization during therapeutic interventions utilizing the horse were collected to suggest PIE as an aid to substance withdrawal and associated cognitive detriments.

The process for this scoping review included 1) the development of five comprehensive research questions, 2) collection of relevant literature from four search engines, 3) team selection of qualifying literature through a multi-step process, 4) charting literature characteristics to determine trends in research, and 5) synthesizing results to predict future practice and research directions.

### 1.5. Development of five comprehensive research questions

To accomplish the aforementioned objectives of this scoping review, the following five questions were developed to scan databases for relevant literature:

1. What are the physical and cognitive implications of substance withdrawal in relation to cortisol?
2. What are the associations between heightened cortisol concentrations and cognitive function?
3. What are the effects of therapeutic interventions utilizing equines on human cortisol and/or heart rate?
4. What are the physiological aspects of heart and cortisol coupling between horses and humans?
5. What are the effects of psychotherapies incorporating human-animal interactions within substance withdrawal therapy?

These questions, along with their qualification criteria, are outlined in Table 1.

### 1.6. Collection of literature through four search engines

The above questions were searched in Google Scholar, PubMed, Medline, and PsychInfo to gather a comprehensive representation of the available literature, including gray literature. Search terms remained constant throughout search engines, though formatting required slight variation between databases (Table 2) and there was no alteration of relevance sorting within search engines.

Search terms were collected from those within Wood et al. [30] including terminology being updated within the industry to ensure publications utilizing outdated terminology associated with equine interaction were not omitted. This selection was intended to retrieve articles most representative of those meeting search criteria within that database. No additional filters were applied to these searches. The first 50 results from each search engine for each question were collected for review, resulting in a possible 200 papers evaluated for each question. Searches were conducted from March of 2022 to January of 2023. This protocol was guided by the PRISMA scoping review protocol [31] and methods documented in Holtcamp et al. [32].

### 1.7. Team selection of qualifying literature through a multi-step process

A team comprised of industry professionals within both the mental health and equine assisted services industry independently reviewed collected papers for qualifying criteria, as outlined in Table 1. If contradiction occurred, deference was given to the senior author of the scoping review. In the first round of this process, it was determined if the titles and abstracts of the collected documents met inclusion criteria. Full articles of selected literature were then evaluated for inclusion criteria. Duplicate articles were removed by the research team. Qualifying articles were considered in this review of literature, as well as contributing information from their references.

### 1.8. Charting literature characteristics to determine trends in research

To evaluate trends in literature surrounding these topics, the following information was gathered from each of the papers: publication year, journal of publication, and key findings. Information collected from research studies in the reviewed literature included: study population, sample size, and physiological and behavioral measures studied. The nature and extent of equine interaction was recorded for studies collected from questions three, four, and five concerning equine studies.

### 1.9. Synthesizing results

To assess PIE as a treatment aid to SUD, particularly during the withdrawal process, the results of this scoping review are reported in a narrative analysis that connects the areas of research. These results consider themes and patterns of the literature, major results, and gaps in research. Literature analysis took place in two phases: 1) a first reading gathering initial themes and information and 2) a second reading in which the aforementioned information was collected and recorded.

## 2. Results & discussion

**Fig. 1.**
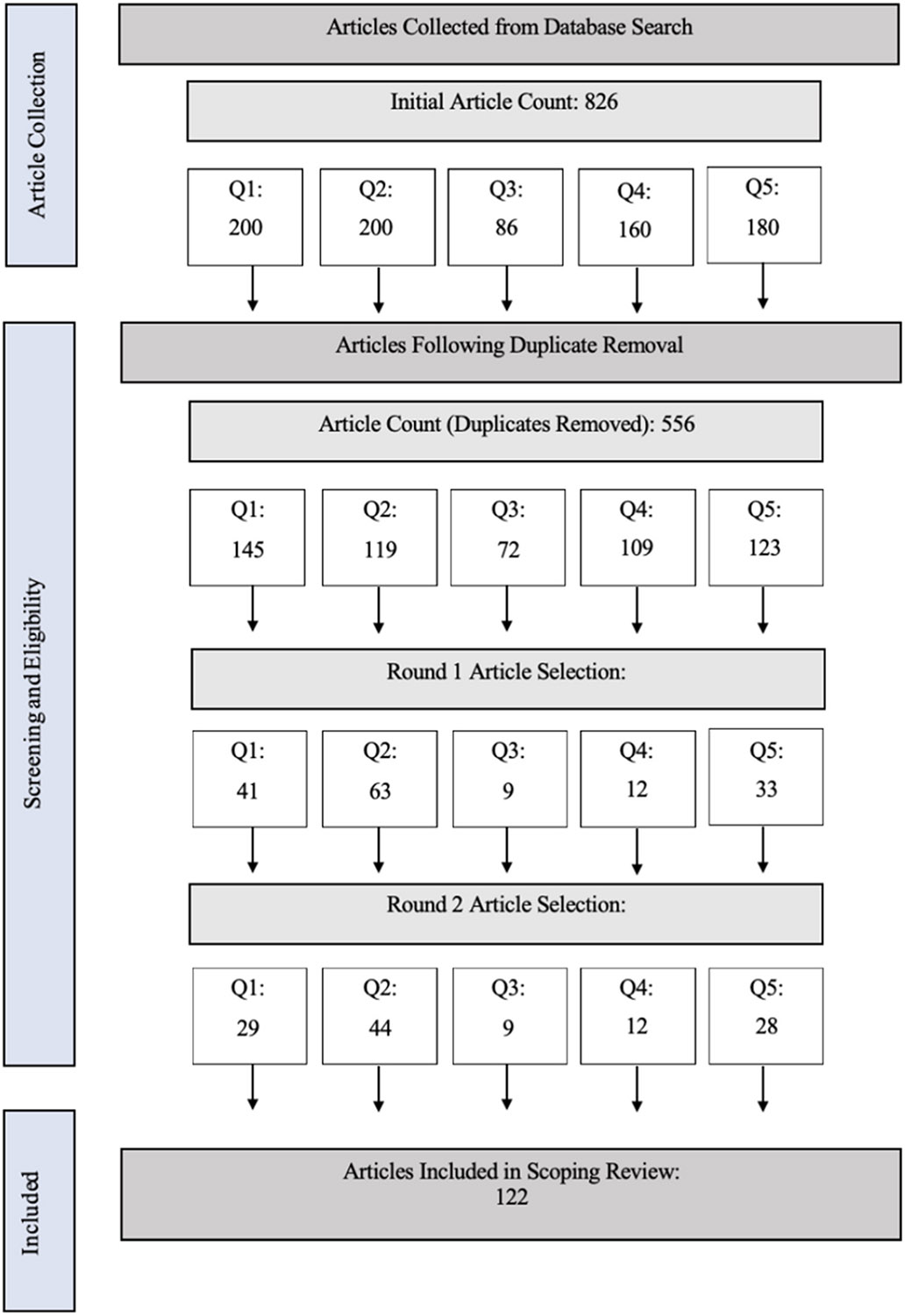
Paper Selection Process during the Scoping Review.

This scoping review is the first to review literature targeting the therapeutic intervention of PIE during the withdrawal process of SUD, particularly as it pertains to cognitive and psychological impacts. In total, 122 publications were included. The process of publication selection is outlined in Fig. 1. Additionally, the publication years, sample sizes, and study populations are outlined in Table 3. While publications addressing all five questions targeted within this review were available, this review highlights areas that could be further explored within the treatment of SUD during withdrawal.

### 2.1. Question 1: what are the physical and cognitive implications of substance withdrawal in relation to cortisol?

The first question of this scoping review identified the role of heightened cortisol in relation to symptoms of substance withdrawal. After duplicate removal, over 100 articles were analyzed to determine this relationship (Fig. 1). Of those articles, 29 were included after multiple rounds of evaluation (Table 1). The resulting articles revealed significant relationships and associations between the well-established rise in cortisol during withdrawal and detrimental physical and cognitive symptoms that could lead to lapses in sobriety [33-37]. Symptoms of note included drug craving, depression and anxiety, memory deficits, attention deficits, executive function deficits, suppressed immune function, and fatigue.

It is well-established that active addiction and substance withdrawal causes hypercortisolism [38-41]. Not only is this stress response important to the specific symptoms of substance withdrawal, but 21 % of studies included suggest it is integral to relapse [14,35,36,40,42,43]. The hypothalamic-pituitary-adrenal (HPA) axis is hyperactivated during drug use and withdrawal, leading to higher basal cortisol concentrations and dulling endocrine stress response effectiveness [40,42,44]. Cortisol secretion interplays with the mesolimbic dopamine system, perpetuating drug use. When cortisol concentrations are altered due to withdrawal, this may lead to relapse and further encourage addiction [14,40,44,45]. As such, 21 % of papers investigating craving reported a positive correlation between craving and cortisol concentrations [33-37]. However, there is some evidence that at relatively low levels of physical stress, cortisol may buffer stress and hinder drug-use related memories, thus, reducing craving [37]. Despite the evidence in the literature reviewed supporting a relationship between cortisol concentrations and substance craving, studies by Huang et al. [46] and Nava et al. [40] failed to find a correlation between cortisol and craving when investigating ketamine and heroin addicts, respectively. Thus, it is apparent that there is a trend in the literature pointing to cortisol’s relationship to craving and relapse, but it may be more complicated than a simple positive correlation.

Aside from the reinforcement of drug use present due to heightened cortisol and dopamine pathways, withdrawal presents a significant challenge due to painful symptoms. Of the literature evaluated, 24 % proposed cortisol is associated with withdrawal severity [14,34,40,44,47-49]. A strict correlation between cortisol concentrations and measures of the Clinical Institute Withdrawal Scale, measuring physical, cognitive, and emotional measures of withdrawal, has been demonstrated under a large variation of treatments [44]. This indicates treatments intended to relieve and mitigate withdrawal severity ought to focus on HPA axis regulation.

The examined literature noted a significant correlation between cortisol and the severity of physical symptoms of withdrawal, such as weakness or fatigue [41,42]. Studies and reviews have also suggested cortisol concentrations during withdrawal may be related to impaired immune function [42,50]. Some studies failed to find a significant relationship between monocyte and plasma cortisol in withdrawal. This is contrary to previous studies, which demonstrate hypercortisolemia impacts leukocyte concentrations. In light of this, authors theorized a slight decrease in cortisol hypersecretion in response to sobriety may have permitted a spike in monocyte circulation, causing results to be at odds with previous literature indicating decreased monocyte circulation in withdrawal [38]. While a correlation between cortisol and symptom severity is well-documented, one study demonstrated treatments that do not affect circulating cortisol can still have a significant impact on withdrawal symptoms. Authors speculated this may have been caused by liver inflammation, as high levels of liver enzymes are also present during withdrawal [50]. This would also suggest an altered immune response within the body due to inflammation. All the effects of irregular cortisol concentrations outlined above, including fatigue, and circulating immune cell variation, may play a role in failed SUD recovery.

Substance withdrawal is also characterized by cognitive deficiencies and emotional difficulties, evident in 80 % of withdrawal patients [51]. Some of these changes have been found to be associated with heightened cortisol. Among the most recognized changes present during withdrawal is depression and anxiety [35,36,41,43,52-56]. These mental health diagnoses disproportionately affect those struggling with hypercortisolemia and withdrawal [53,56], and these all related back to cortisol concentrations within the body.

An important component of SUD recovery is the development of healthy habits to replace habitual compulsions of addiction. As such, the effect of cortisol on cognitive abilities and memory are of concern in the process of recovery. Research regarding the impact of withdrawal on learning and/or memory reveals diminished memory and problem-solving related to logical, visual, and verbal memory significantly correlated with cortisol concentrations when investigated separately [15,33]. While the specific types of memory affected are examined extensively in the following section of this review, it’s important to note at this point in the review that impacts to all cognitive domains present a significant challenge to SUD recovery.

### 2.2. Question 2: what are the associations between heightened cortisol concentrations and cognitive function?

While research investigating the effects of hypercortisolism in withdrawal has been conducted in some depth, few studies delve into types of cognition most affected in this state. Understanding the effects of hypercortisolism on specific cognitive functions is necessary to determine specific cognitive deficits withdrawal patients may be experiencing. Working memory and executive functions are especially important to the success of SUD recovery, and thus, detriments to these systems are important to note and attempt to relieve. To investigate this area of research within this scoping review, over 100 articles were initially selected, but 44 were included after inclusion and exclusion criteria were evaluated (Fig. 1, Table 3).

As mentioned previously, the physical and/or cognitive impacts of abnormal cortisol concentrations can be characterized as an inverted U-shape relationship, meaning excessively low concentrations of cortisol as well as chronically high concentrations have detrimental effects [15,57,58]. These effects are likely due to the occupation of both glucocorticoid receptors (GRs) and mineralocorticoid receptors (MRs). Cortisol interacts and binds to both types of receptors but has a higher affinity for MRs during times of basal cortisol concentrations and begins to occupy GRs during times of increased cortisol circulation [15]. These receptors are especially prevalent in the hippocampus and the prefrontal cortex, respectively. These areas are important for memory retrieval and responsible for many of the cognitive functions as outlined in this review [18,59].

A wide variation of memory was analyzed throughout resulting literature, but there was a prominent trend towards the impact of cortisol on explicit memory with 72 % of the included literature investigating this measure. Explicit memory, also known as declarative memory, is an important long-term memory function consisting of information one can consciously recall and express [60]. Types of explicit memory include the following: 1) episodic memory, in which long-term memories of specific events are held; 2) sematic memory, relating to facts, concepts, and names; and 3) spatial memory, which is responsible for tracking and memory of locations and objects [61]. The alternative to explicit memory is implicit memory, referring to unconsciously retained information and includes procedural memories that can be reflected in the performance of a task [60].

A majority of studies included (61 %) measured cognitive function relating to executive function. Executive function consists of planning, working memory, inhibition, concept formation, and set-shifting of working memory, flexible thinking, and self-control skills, and as such can be measured by tests of working memory, set-shifting, inhibition, and concept formation. Specifically, working memory is the function immediately following the intake of information and refers to the ability to hold new information briefly before sorting it for storage or connecting with other relevant information [12]. Set shifting refers to the ability to shift attention to new information [62,63]. Both are valuable executive functions that have been studied as to the relationship with that of substance abuse.

The executive function working memory was heavily studied, investigated by 43 % of the literature. It was predominantly reported that working memory is impaired in the presence of elevated cortisol [18,28,58,63-68]. This is likely due to the prevalence of GRs in the prefrontal cortex, where working memory and executive functions are highly regulated [68]. The occupation of MRs in the hippocampus at basal cortisol concentrations would not impair working memory, but when excess cortisol occupies GRs in the prefrontal cortex, working memory and executive functions present in that region are impaired. Therefore, executive functions are at odds with the U-shaped trend seen with other types of memory. Instead, cortisol and executive memory have a linear relationship [68]. Of studies investigating the impact of cortisol concentrations on working memory, 57 % found a significant relationship between cortisol and working memory, even when impacts on other cognitive parameters proved insignificant [58,62,67,69]. Thus, working memory parameters may be more sensitive to stress detriments than other types of memory.

Attention contributes to working memory and studies tracking this aspect found significant deficiencies in the presence of increased cortisol as well [18,28,69-73]. Interestingly, studies investigating set-shifting, or the flexibility of attention, did not demonstrate a significant relationship within the investigated literature [28,62,63,74,75]. As such, it may be the case that hypercortisolism affects working memory and attention measures to a greater extent than other cognitive functions, while less extensively impacting set-shifting abilities. However, hypercortisolism is still commonly cited as a main contributor of sobriety struggles, as executive function and working memory deficits hinder the reasoning and self-control within the prefrontal cortex that encourage choices of sobriety.

A majority of the literature (59 %) examined declarative memory impairments, and effects of cortisol on these functions were varied. Of these papers, 58 % reported significant associations between heightened cortisol and verbal or declarative memory [18,70]. Though the reported effects of heightened cortisol on explicit memory vary, it appears 72 % of studies that failed to find a correlation were investigating healthy, relatively young populations [28,67,73,76]. Alternatively, studies that reported significant relationships between these measures investigated depressed, elderly, schizophrenic, substance-dependent, and otherwise compromised populations [18,70,76-82]. Thus, it appears cortisol concentrations must exceed a certain threshold not met in healthy subjects to impact declarative memory in a notable manner.

Cortisol was reported to account for 5–16 % of cognitive variance and was a strong predictor of high memory test variance [83]. These impacts are seen especially to the detriment of visuospatial and declarative memory. Working memory and executive function have a negative linear relationship with cortisol, as these functions are impaired by the occupation of GRs in the prefrontal cortex, which is otherwise unaffected by MR occupation. The effects of cortisol on working memory and executive function are especially concerning in SUD recovery, as the absence of such skills makes addicts especially susceptible to relapse through habitual use.

### 2.3. Question 3: what are the effects of therapeutic interventions utilizing equines on human cortisol and/or heart rate?

The third question of this scoping review investigated the impacts of equine interaction within psychotherapy on stress responses outlined above to suggest this interaction as a means to alleviate responses of stress, and thus, cognitive impacts. Despite the potential for 200 papers to be collected for evaluation of this topic, only 86 papers resulted from the search, and of that only 9 fit the simple inclusion criteria of evaluating basic physiological responses to equine interaction for psychological purposes (Fig. 1, Table 3). This points to a noteworthy gap in the literature associated with this form of a therapeutic intervention.

Despite the growth of equine interaction within the psychological treatment space, this therapeutic approach lacks substantiated evidence pointing to physical benefits. Of literature evaluated, 11 % featured exclusively anecdotal, qualitative observations of the author’s experience with human-equine interaction within psychotherapy [84]. Within the 8 papers relating to the physiological stress responses to equine interaction, 66 % of the interactions included explicitly intentional therapeutic benefits [25,85-89]. The remaining articles recognized benefits of equine interaction without inclusion of therapeutic interventions. Furthermore, only 44 % of literature cited compliance with a recognized equine assisted services association such as the Professional Association of Therapeutic Horsemanship International (PATH Intl.) or Equine Assisted Growth and Learning Association (EAGLA) [22,86,88,89]. These associations indicate the required presence of a mental health professional within the treatment setting when labeling the equine interaction as being therapeutic and a part of the psychotherapy process. Therapy utilizing the horse as a treatment tool differs from everyday human-equine interactions through adherence to intentional conversations as determined and carried out by a mental health professional [30]. The scarcity of such cases in the literature reflects a larger industry misunderstanding leading to the dismissal of human-equine interaction as a therapeutic tool within the mental health community.

Further limitation of literature in this area are sample sizes and treatment quantities. The literature examined had an average sample size of 23 individuals participating in interventions utilizing equines (Table 3). The sample sizes were as small as 4 [23] and as large as 64 [88]. Sample size challenges are attributed to limited horse availability, locations, and patients and mental health professionals willing to participate within the scientific process of the studies. Despite limited sample sizes, there was evidence of decreased cortisol concentrations in participants with populations ranging from healthy children, those with post-traumatic stress disorder (PTSD), and those on the autistic spectrum [23,88,89]. These reductions in cortisol concentrations were accompanied by other positive impacts such as reports of increased positive emotion as reported in Pendry et al. [88]. Reductions in human heart rates were also noted in 33 % of studies, even when cortisol reduction failed to be affected [87]. This may indicate that heart rate levels are more easily affected, but still points to a calming effect present during the equine interaction.

While three studies included in this review failed to find significant reductions in human cortisol concentrations or heart rates within equine therapeutic interaction, three of the nine papers included in this question found synchronization of heart rate or cortisol concentrations between equine and human participants [23,25,87]. This synchronization may indicate an important relationship between humans and horses during therapy-based interaction. In Yorke et al. [23], four children with PTSD participated in six hour-long therapeutic sessions incorporating the horse. During these sessions, researchers found there was “mild to moderate” cortisol level symmetry occurring between children and horse pairs. This interaction in a short time frame with a significantly stressed population suggests similar investigations in populations such as recovering addicts, where a physiological connection with a horse’s lower cortisol concentrations could prove grounding. In Baldwin et al. [25], heart rate synchronization in eight of thirteen human-horse pairs occurred during just four sessions of ten-minute guided therapeutic interactions. Similar findings were reported by Naber et al. [87] between intellectually disabled clients interacting with their favorite therapy horse in two twenty-minute sessions, although it was not reported in those interacting with a random horse. Thus, more research is needed to determine the familiarity between horse and human required to elicit physiological synchronization within a therapeutic setting.

### 2.4. Question 4: what are the physiological aspects of heart and cortisol coupling between horses and humans?

Research interests have grown concerning human-equine interaction surrounding the mystery of herd-bound prey animals permitting close, trusting relationships with a predatory species. This phenomenon coupled with anecdotes about calming effect of horses has led researchers to question the contribution of physiological interactions between humans and horses to this relationship, particularly regarding heart rate and cortisol. Despite the value of this physiological relationship within a therapeutic setting as observed within the previous question of this review, researchers have broadened the scope of this investigation to various types of interaction between human and horse including that outside of the therapeutic environment. Nevertheless, even with the expanded scope for question 4 beyond the therapeutic setting, the investigation of synchronizations between humans and horses during various forms of interaction is relatively new, with pertinent studies appearing just 12 years ago. Further, a search of 160 papers uncovered just 12 publications that investigated specifically physiological coupling during human-equine interactions (Fig. 1, Table 3). Within this limited field of study, 25 % of these studies appeared in the gray literature, as this emerging topic is only beginning to appear in reputable research. Even within this small number of studies, the nature of human-equine interaction, the length of the interaction, and the results of the interaction have been highly variable. Interactions included manufactured ground-based equine-interaction (41 %), un-mounted therapeutic interaction (17 %), mounted therapeutic interaction (17 %), and riding lessons (8 %). Study durations ranged from less than one day (58 %), between one day and one week (8 %), one week and six weeks (17 %), and over 11 weeks (17 %).

The results of these studies spanned from no apparent physiological coupling between humans and horses (33 %) to the majority of participants coupling with equine counterparts (12 %). Most variation in results appeared in studies analyzing heart rate interactions. While 75 % of included studies tracking cardiovascular coupling effects found a positive correlation, the remaining studies failed to find a relationship. These studies lacking coupling included equine-interaction limited to a short, manufactured problem-solving and stress-inducing challenges for the purpose of creating interaction exclusively for research [90,91]. However, one study did reveal heart coupling correlation despite the research-based manufactured human-equine interaction in an environment more closely related to everyday horse management [92]. This study indicated contact initiation between humans and horses decreased coupling as opposed to exclusively visual or olfactory contact. In the sphere of equestrian sports, Bridgeman [93] uncovered a significant positive relationship between the heart rates of humans and their horses in the training environment of a show-jumping competition. Within a therapeutic setting, all studies investigating heart rate interactions found positive correlations of varying strengths and frequencies within populations, pointing to the quantitative efficacy of equine interaction [25,28,93,94].

In the case of cortisol coupling, studies investigating interactions found significant correlation between humans and horses in all three studies [23,90,95]. In Peeters et al. [95], group means of horses and their owners’ salivary cortisol measures when competing in a show-jumping competition were strongly correlated. However, Yorke et al. [23] found a “mild to moderate” correlation between human and horse salivary cortisol when investigating coupling in a therapeutic setting between children with PTSD and horses. Similarly, a study investigating cortisol coupling in a riding lesson setting found humans and horse cortisol concentrations were correlated surrounding a two-hour lesson. Horses’ cortisol concentrations prior to the lesson were positively correlated with riders’ cortisol concentrations following the lesson, pointing to an interaction between horse and rider [90]. Thus, it appears that cortisol coupling may occur even if human and horse participants have no prior relationship, though a relationship may strengthen the cortisol coupling interaction, although further research is necessary to completely understand the coupling within cortisol between human and horse.

### 2.5. Question 5: what are the effects of psychotherapies incorporating human-animal interactions within substance withdrawal therapy?

With the influence animals have on physiological symptoms, therapeutic inventions incorporating animal interaction are emerging as a means of SUD recovery. Similar to other questions investigating the use of animals as a therapeutic tool and associated benefits of human-animal interaction, this research question is in its beginning stages with only 28 relevant papers included in this review (Fig. 1). Studies included in this question were all published in the last 24 years (Table 3). Additionally, the novelty of this research question is reflected in a lack of substantiated scientific articles surrounding the topic. Only 60 % of the included studies were therapeutic interventions utilizing animals as a treatment tool where effects were recorded for the SUD populations. The remaining studies were literature reviews, descriptions associated with animals utilized as a therapeutic intervention, or narrative portrayals of human-animal interaction within a therapeutic setting and its impacts. Furthermore, none of the publications included in this question measured physiological values nor did they target the withdrawal stage of SUD recovery. All publications measured behavioral measurements or presented literature reviews, predominantly utilizing self-reported surveys (43 %) and semi-structured interviews (39 %). Only 14 % of studies noted statistical significance to their results. These observations of the literature indicate a strong need for quantitative, reliable accounts of the impact of therapeutic animal interactions on SUD recovery.

Although no physiological evidence was presented in the literature, there were common themes of impact through self-reported or narrative accounts that appeared throughout the literature. A theme within 29 % of publications was the improvement of the therapeutic alliance with the use of animal interaction within the therapeutic setting [96-99]. Wesley et al. [99] found that in a population of 136 SUD recovery patients, the use of animal interaction improved participants’ perception of the therapeutic alliance. In a study investigating the impact of canine interaction on eight SUD recovering patients, Campbell-Begg [97] found just the presence of a dog in sessions allowed nurses and participants to improve communication, paving the way for quality therapeutic interactions. Sikstrom et al. [98] investigated the use of canine interaction in “hard to reach” populations that included participants with social disadvantages, communication barriers, and power imbalances, finding the therapeutic setting created a rapport between participants and therapists. Some participants noted that the dogs made them feel more humanized and decreased their anxiety in interaction, acting as a buffer.

The therapeutic alliance is affected by a number of factors, and there are a number of reasons human-animal interaction demonstrated a positive impact. The comfort and confidence of patients when interacting with their therapist is a significant contribution, and one the literature reported animal interaction improved. Kern-Godal et al. [100] found the use of human-equine interaction gave patients a sense of purpose and identity, allowing them to step outside of their role as patient to complete activities that gave them the feeling of “doing something useful.” This feeling of improved identity was seen in 18 % of studies as well and may contribute to patient comfort, confidence, and retention. Anxiety and depression may also hinder feelings of comfort and confidence, so it is notable that 25 % of studies found human-animal interaction worked to reduce feelings of anxiety and depression in therapy [24,96,101-104]. A study using self-reporting surveys determined that participants of canine interaction experienced significant reductions in feelings of anxiety [105]. Furthermore, a study measuring the impact of canine interaction via self-reporting symptom checklists and surveys found significant improvement in participant social skills, which they credited with decreased feelings of depression, anxiety, and craving [102]. This was the only study to question craving, but Uhlmann et al. [106] observed a decreased frequency of smoking in their SUD population when investigating implementation of canine interaction. Measurements of these values could be beneficial in future research with the addition of more physiologically-based measurements to substantiate such findings.

As for the impact as it relates to soft skills, 29 % of studies found improvements in such things as communication within SUD populations [24,101,107-112]. These soft skills may help patients better interact with therapists to improve treatment experience as well as form other important relationships to support recovery. Atherton et al. [24] measured the impact of equine interaction within the therapeutic setting finding an increase in social skills such as support, acceptance, listening, trust, and respect as well as a decrease in depressive and anxious behaviors. Bark [101] revealed that teenage participants demonstrated increased focus, appeared happier, and were more communicative with the use of equine interaction within the treatment. These improvements in mental health, social, and emotional skills can improve SUD patients’ therapeutic experience and encourage them to be more receptive to treatment.

Some of the literature (18 %) credited the success of therapeutic interventions incorporating animal interactions with behaviors of the animals that elicited desirable responses in patients. Furthermore, 11 % of papers noted the animals provided SUD patients an outlet for safe and healthy touch [99,107,110,113-115]. This may have contributed to patients’ overall comfort and built confidence in the safety of the interaction. Specifically, in equine interventions, a natural response of the horse that had a positive impact on patients’ treatment was the role of the horse as a biofeedback machine [96,103]. This refers to the way in which horses as a herd prey animal visibly react and respond to patients’ actions [116]. This provides important information to therapists as well as opportunities for patients and therapists to alter behavior and observe the impacts.

A major challenge in SUD recovery is patient retention, particularly during the withdrawal process. Due to the apparent mental, emotional, and social benefits human-equine interaction offers, this treatment is a likely solution to retention challenges. Kern-Godal et al. [21] found a significant association in a 56 % completion rate of young adults in SUD treatment supplemented with equine interaction, as opposed to a 14 % completion rate in those that did not receive this interaction within the treatment process. While improved retention rate was noted in 22 % of the literature, this result was not seen in all studies. Gatti et al. [117] found no significant impact of equine interaction on the retention rate of 37 SUD recovering patients. However, this outcome may be due to the practices of the individual treatment center. This is reflective of the discrepancy varied practices within therapeutic treatment options utilizing animal interventions.

## 3. Conclusions

The troubling rise in SUD within the United States over the past few years requires treatment program advancement to improve treatment responses, recovery rates, and longevity of sobriety. This scoping review established the impacts addiction and recovery have on cortisol concentrations, the consequence of heightened cortisol on cognitive function integral to recovery, and the benefits of human-equine interaction within a therapeutic setting as demonstrated by psychological measures. In addition, this review offered research supporting physiological coupling between humans and horses. Literature within this review further detailed the impact of chronically high cortisol on executive function, which can hinder SUD patients from forming new, healthy sober habits, particularly during the withdrawal process. The ability of equine interaction within the therapeutic setting to decrease cortisol, perhaps through physiological coupling, suggests PIE may be a valuable treatment method to mitigating physiological difficulties during the recovery process, especially during withdrawal. Existing literature on equine interaction within SUD recovery revealed improved retention rates, therapeutic alliances, patient comfort, confidence, mental health, emotional stability, and communication skills. Nevertheless, as seen through this review, there is little research targeting substance withdrawal, and what is available lacks quantitative analysis with large sample sizes. As such, future research efforts should focus on investigating the impact of standardized practices for PIE on physiological parameters utilizing increased sample sizes of SUD patients where progression of the treatment process is tracked including the initial stages of the treatment period when the patient is actively going through substance withdrawal.

## Figures and Tables

**Table 1:** Scoping Review Questions and Qualifications.

Question 1: What are the physical and cognitive implications of substance withdrawal in relation to cortisol?	
Inclusion	Exclusion
Describes withdrawal symptoms in relation to increased cortisol levels	Withdrawal from substances not notable for the purposes of this review, including nicotine, tobacco, cannabis, and caffeine
Measured cortisol response in relation to, or in conjunction with, withdrawal symptoms	Medical drug use withdrawal (e.g., steroids or glucocorticoids used in treatment)
Cortisol as a predictor of addiction relapse or withdrawal severity during the withdrawal period	Effects of cortisol associated exclusively with active addiction, not including withdrawal period
	Only withdrawal symptom in question is cortisol levels
Question 2: What are the associations between heightened cortisol levels and cognitive function?	
Cortisol/glucocorticoid impact on cognition, with the inclusion of specific or various types of memory or cognition (e.g., verbal cognition, episodic memory, etc.)	Cortisol/glucocorticoid impact on global cognition or memory as a whole without breakdown into specific types of memory or cognition
Impact of glucocorticoid or mineralocorticoid receptors on specific or various types of memory or cognition	Discussion limited to HPA axis function without specific discussion of cortisol
	Discussion concerning cortisol replacement therapy
Question 3: What are the effects of therapeutic interventions utilizing equines on human cortisol and/or heart rate?	
Equine therapy, equine assisted therapy, equine assisted psychotherapy, equine assisted learning, or equine assisted services with a focus on equine assisted interventions as a means of aid to mental health or neurologic benefits	Hippotherapy, therapeutic horse riding, medical treatment of horses, or alternative animal therapies
	Equine assisted interventions for purely physical goals
Question 4: What are the physiological aspects of heart and cortisol coupling between horses and humans?	
Correlations, synchronizations, and similarities in human-equine heart rate	Regular circadian rhythms or variations in human or equine heart rates and/or cortisol levels
Correlations, synchronizations, and similarities in human-equine cortisol levels	Human or equine reactions to the other without physiological basis in cortisol or heart rate
	Medical- or exercise-induced changes in heart rate and/or cortisol in humans or horses
Question 5: What are the effects of psychotherapies incorporating human-animal interactions within substance withdrawal therapy?	
Physiological responses to psychotherapies incorporating animal interactions for substance withdrawal patients	Responses to psychotherapies incorporating animal interactions by those experiencing alternative infections, diseases, health complications, or medical intervention withdrawal
Psychological responses to psychotherapies incorporating animal interactions for substance withdrawal patients	Substances involved are limited to nicotine, tobacco, cannabis, and caffeine
	Responses to human-animal interactions that are limited to self-reported effects

**Table 2:** Database Search Terms.

Question 1: What are the physical and cognitive implications of substance withdrawal in relation to cortisol?	
Database	Search Terms
Google Scholar	Withdrawal cortisol symptom syndrome OR drug OR alcohol -tobacco -cannabis -smoking
PubMed	(Substance withdrawal OR withdrawal syndrome OR drug withdrawal OR alcohol withdrawal AND (cortisol) AND (symptoms) NOT (tobacco cannabis smoking)
Medline	(Substance withdrawal OR withdrawal syndrome OR drug withdrawal OR alcohol withdrawal AND (cortisol) AND (symptoms) NOT (tobacco cannabis smoking)
PsychInfo	(Substance withdrawal OR withdrawal syndrome OR drug withdrawal OR alcohol withdrawal AND (cortisol) AND (symptoms) NOT (tobacco cannabis smoking)
Question 2: What are the associations between heightened cortisol levels and cognitive function?	
Google Scholar	Cortisol glucocorticoid memory cognition cognitive function
PubMed	(Cortisol OR glucocorticoid) AND (memory AND cognition AND cognitive function)
Medline	(Cortisol OR glucocorticoid) AND (memory AND cognition AND cognitive function)
PsychInfo	(Cortisol OR glucocorticoid) AND (memory AND cognition AND cognitive function)
Question 3: What are the effects of therapeutic interventions utilizing equines on human cortisol and/or heart rate?	
Google Scholar	(Cortisol OR heart rate) AND (equine psychotherapy OR equine assisted therapy OR equine assisted learning OR equine assisted services) NOT (therapeutic riding) NOT hippotherapy
PubMed	(Cortisol OR heart rate) AND (equine psychotherapy OR equine assisted therapy OR equine assisted learning OR equine assisted services) NOT (therapeutic riding) NOT (hippotherapy)
Medline	(Cortisol OR heart rate) AND (equine psychotherapy OR equine assisted therapy OR equine assisted learning OR equine assisted services) NOT (therapeutic riding) NOT (hippotherapy)
PsychInfo	(Cortisol OR heart rate) AND (equine psychotherapy OR equine assisted therapy OR equine assisted learning OR equine assisted services) NOT (therapeutic riding) NOT (hippotherapy)
Question 4: What are the physiological aspects of heart and cortisol coupling between horses and humans?	
Google Scholar	(Heart OR cortisol) AND (synchronize OR couple OR correlation) AND (horse OR equine) AND (human OR rider OR person)
PubMed	(Heart OR cortisol) AND (synchronize OR couple OR correlation) AND (horse OR equine) AND (human OR rider OR person)
Medline	(Heart OR cortisol) AND (synchronize OR couple OR correlation) AND (horse OR equine) AND (human OR rider OR person)
PsychInfo	(Heart OR cortisol) AND (synchronize OR couple OR correlation) AND (horse OR equine) AND (human OR rider OR person)
Question 5: What are the effects of psychotherapies incorporating human-animal interactions within substance withdrawal therapy?	
Google Scholar	(Animal assisted therapy OR animal assisted psychotherapy OR animal assisted learning) AND (substance OR alcohol OR drug) AND (addiction OR withdrawal)
PubMed	(Animal assisted therapy OR animal assisted psychotherapy OR animal assisted learning) AND (substance OR alcohol OR drug) AND (addiction OR withdrawal)
Medline	(Animal assisted therapy OR animal assisted psychotherapy OR animal assisted learning) AND (substance OR alcohol OR drug) AND (addiction OR withdrawal)
PsychInfo	(Animal assisted therapy OR animal assisted psychotherapy OR animal assisted learning) AND (substance OR alcohol OR drug) AND (addiction OR withdrawal)

**Table 3:** Publications included for the Scoping Review Questions.

Question	Range of Publication Year	Mean Sample Size of Studies In Question	Populations Studied
1	1979 – 2021	36.75	Alcohol-dependent (n = 14); Alcohol-induced pseudo-Cushings (n = 1); Literature Review (n = 5); Heroin-Dependent (n = 3); Opiate-Dependent (n = 2); Polysubstance misuse (n = 1); Morphine-Dependent (n = 2); Alcohol-Exposed (n = 1); Ketamine-Dependent (n = 1);
2	1996 – 2022	52.53	Literature Review or Metanalysis (n = 8); Depressed (n = 4); College Students (n = 1); Healthy (n = 20); Children (n = 2); Elderly (n = 3); Schizophrenic (n = 3); Cocaine-Dependent (n = 1); Burnout (n = 1); Anorexia Nervosa (n = 1); Non-human Subjects (n = 1); Cancer Survivors (n = 1); Depressed (n = 1); Cushings (n = 1)
3	2012 – 2021	23.13	Children (n = 5); Healthy (n = 2); Intellectually Disabled (n = 1); At-Risk (n = 1); Autistic (n = 1); PTSD (n = 2); Elderly (n = 1); Narrative Review (n = 1)
4	2009 – 2021	16.67	Children (n = 2); College Students (n = 2); PTSD (n = 1); Healthy (n = 8); Elderly (n = 1); SUD Recovering (n = 1)
5	1998 – 2022	37.35	SUD Recovering (n = 18); Youth/Young Adults (n = 8); At-Risk (n = 1); Literature Review (n = 6); Accounts of Programs (n = 2); Psychiatric (n = 2)

## References

[1] Understanding the Epidemic. (2021). Centers for Disease Control and Prevention. Retrieved April 3, 2023, from 〈https://www.cdc.gov/drugoverdose/epidemic/index.html〉.

[2] Bustamante J, NCDAS, Subst. Abus. Addict. Stat (2022). 〈https://drugabusestatistics.org/〉.

[3] Abramson A. (2021). Substance use during the pandemic. Monitor on Psychology. 〈https://www.apa.org/monitor/2021/03/substance-use-pandemic〉.

[4] Hser Y-I, Evans E, Huang D, Anglin DM, Relationship between drug treatment services, retention, and outcomes, Psychiatr. Serv 55 (2004) 767–774, 10.1176/appi.ps.55.7.767. 15232015

[5] Fort Behavioral Health. (2020). Why dropping out of treatment is so prevalent and what we can do about it. Fort Behavioral Health Retrieved from 〈https://www.fortbehavioral.com/addiction-recovery-blog/why-dropping-out-of-treatment-is-so-prevalent-and-what-we-can-do-about-it/〉.

[6] Hoseinie L, Gholami Z, Shadloo B, Mokri A, Amin-Esmaeili M, Rahimi-Movaghar A, Drop-out from a drug treatment clinic and associated reasons, East. Mediterr. Health J 23 (2017) 173–181, 10.26719/emhj.17.018. 28493264

[7] Tiffany ST, A cognitive model of drug urges and drug-use behavior: Role of automatic and nonautomatic processes, Psychol. Rev 97 (1990) 147–168, 10.1037/0033-295X.97.2.147. 2186423

[8] American Psychiatric Association. Diagnostic and statistical manual of mental disorders, 5th ed.)., American Psychiatric Publishing, 2013.

[9] Österberg K, Karlson B, Malmberg B, Hansen ÅM, A follow-up of cognitive performance and diurnal salivary cortisol changes in former burnout patients, Stress 15 (2012) 589–600, 10.3109/10253890.2012.665435. 22168599

[10] Ornstein T, Profiles of cognitive dysfunction in chronic amphetamine and heroin abusers, Neuropsychopharmacology 23 (2000) 113–126, 10.1016/S0893-133X(00)00097-0. 10882838

[11] Rogers RD, Robbins TW, Investigating the neurocognitive deficits associated with chronic drug misuse, Curr. Opin. Neurobiol 11 (2) (2001) 250–257, 10.1016/S0959-4388(00)00205-4. 11301247

[12] Baddeley A, Working memory, Science 255 (1992) 556–559, 10.1126/science.1736359. 1736359

[13] Miller R, Weckesser LJ, Smolka MN, Kirschbaum C, Plessow F, Hydrocortisone accelerates the decay of iconic memory traces: On the modulation of executive and stimulus-driven constituents of sensory information maintenance, Psychoneuroendocrinology 53 (2015) 148–158, 10.1016/j.psyneuen.2015.01.008. 25618593

[14] Bearn J, Buntwal N, Papadopoulos A, Checkley S, Salivary cortisol during opiate dependence and withdrawal, Addict. Biol 6 (2001) 157–162, 10.1080/13556210020044403. 11341855

[15] Erickson K, Drevets W, Schulkin J, Glucocorticoid regulation of diverse cognitive functions in normal and pathological emotional states, Neurosci. Biobehav. Rev 27 (2003) 233–246, 10.1016/S0149-7634(03)00033-0. 12788335

[16] Domes G, Rothfischer J, Reichwald U, Hautzinger M, Inverted-U function between salivary cortisol and retrieval of verbal memory after hydrocortisone treatment, Behav. Neurosci 119 (2) (2005) 512–517, 10.1037/0735-7044.119.2.512. 15839797

[17] Takahashi T, Ikeda K, Ishikawa M, Tsukasaki T, Nakama D, Tanida S, Kameda T, Social stress-induced cortisol elevation acutely impairs social memory in humans, Neurosci. Lett 363 (2004) 125–130, 10.1016/j.neulet.2004.03.007. 15172099

[18] Hinkelmann K, Moritz S, Botzenhardt J, Riedesel K, Wiedemann K, Kellner M, Otte C, Cognitive impairment in Major Depression: Association with circadian salivary cortisol, Pharmacopsychiatry 42 (2009), 10.1055/s-0029-1220738. 19709646

[19] Jameison K, Dinan TG, Glucocorticoids and cognitive function: From physiology to pathophysiology, Hum. Psychopharmacol.: Clin. Exp 16 (2001) 293–302, 10.1002/hup.286. 12404564

[20] Wingenfeld K, Otte C, Mineralocorticoid receptor function and cognition in health and disease, Psychoneuroendocrinology 105 (2019) 25–35, 10.1016/j.psyneuen.2019.02.024. 30243757

[21] Kern-Godal A, Arnevik EA, Walderhaug E, Ravndal E, Substance use disorder treatment retention and completion: A prospective study of horse-assisted therapy (HAT) for young adults, Addict. Sci. Clin. Pract (2015) 10, 10.1186/s13722-015-0040-5. PMC4672500 26466788

[22] Malinowski K, Yee C, Tevlin JM, Birks EK, Durando MM, Pournajafi-Nazarloo H, Cavaiola AA, McKeever KH, The effects of equine assisted therapy on plasma cortisol and oxytocin concentrations and heart rate variability in horses and measures of symptoms of post-traumatic stress disorder in veterans, J. Equine Vet. Sci 64 (2018) 17–26, 10.1016/j.jevs.2018.01.010. 30973147

[23] Yorke J, Nugent W, Strand E, Bolen R, New J, Davis C, Equine-assisted therapy and its impact on cortisol concentrations of children and horses: A pilot study and meta-analysis, Early Child Dev. Care 183 (2013) 874–894, 10.1080/03004430.2012.690703.

[24] Atherton WL, Meola CC, Pritchard KS, Innovative equine facilitated psychotherapy intervention for adolescent addiction treatment: A pilot study, Int. J. High. Risk Behav. Addict 9 (1) (2020) e99013, 10.5812/ijhrba.99013.

[25] Baldwin A, Rector B, Alden A, Physiological and behavioral benefits for people and horses during guided interactions at an assisted living residence, Behav. Sci 11 (2021) 129, 10.3390/bs11090129. 34677222 PMC8533143

[26] Handlin L, Nilsson A, Ejdebäck M, Hydbring-Sandberg E, Uvnäs-Moberg K, Associations between the psychological characteristics of the human–dog relationship and oxytocin and cortisol concentrations, AnthrozoöS. 25 (2012) 215–228, 10.2752/175303712X13316289505423.

[27] Keeling LJ, Jonare L, Lanneborn L, Investigating horse-human interactions: The effect of a nervous human, Vet. J 181 (2009) 70–71, 10.1016/j.tvjl.2008.03.013. 19394879

[28] Lanata A, Guidi A, Valenza G, Baragli P, & Scilingo EP (2016). Quantitative heartbeat coupling measures in human-horse interaction. 2016 38th Annual International Conference of the IEEE Engineering in Medicine and Biology Society (EMBC). https://doi.org/10.1109. 10.1109/EMBC.2016.7591286 28268877

[29] Scopa C, Contalbrigo L, Greco A, Lanatà A, Scilingo EP, Baragli P, Emotional transfer in human–horse interaction: New perspectives on equine assisted interventions, Animals 9 (2019) 1030, 10.3390/ani9121030. 31779120 PMC6941042

[30] Wood W, Alm K, Benjamin J, Thomas L, Anderson D, Pohl L, Kane M, Optimal Terminology for Services in the United States That Incorporate Horses to Benefit People: A Consensus Document, J. Altern. Complement. Med. (N. Y., N. Y.) 27 (1) (2021) 88–95, 10.1089/acm.2020.0415. 33252244

[31] Tricco AC, Lillie E, Zarin W, O’Brien KK, Colquhoun H, Levac D, Moher D, Peters MDJ, Horsley T, Weeks L, Hempel S, Akl EA, Chang C, McGowan J, Stewart L, Hartling L, Aldcroft A, Wilson MG, Garritty C, Lewin S, Godfrey CM, Macdonald MT, Langlois EV, Soares-Weiser K, Moriarty J, Clifford T, Tunçalp Ö, Straus SE, Prisma extension for scoping reviews (PRISMA-SCR): Checklist and explanation, Ann. Intern. Med 169 (2018) 467–473, 10.7326/M18-0850. 30178033

[32] Holtcamp K, Galarneau KD, Nicodemus M, Phillips T, Christiansen D, Rude B, Ryan PL, Scoping review of the role of equine assisted psychotherapy and learning in opioid abuse treatment, J. Vet. Behav 74 (2024) 1–10, 10.1016/j.jveb.2024.06.010.

[33] Fathi D, Abulsoud AI, Saad MA, Nassar NN, Maksimos MM, Rizk SM, Senousy MA, Agomelatine attenuates alcohol craving and withdrawal symptoms by modulating the Notch1 signaling pathway in rats, Life Sci. 284 (2021) 119904, 10.1016/j.lfs.2021.119904. 34453945

[34] Keedwell PA, Poon L, Papadopoulos AS, Marshall EJ, Checkley SA, Salivary cortisol measurements during a medically assisted alcohol withdrawal, Addict. Biol 6 (2001) 247–257, 10.1080/13556210120055860. 11900603

[35] Milivojevic V, Angarita GA, Hermes G, Sinha R, Fox HC, Effects of prazosin on provoked alcohol craving and autonomic and neuroendocrine response to stress in alcohol use disorder, Alcohol.: Clin. Exp. Res 44 (2020) 1488–1496, 10.1111/acer.14374. 32449942 PMC7572699

[36] Shi J, Li S.-x, li Zhang X, Wang X, Foll BL, Zhang X-Y, Kosten TR, Lu L, Time-dependent neuroendocrine alterations and drug craving during the first month of abstinence in heroin addicts, Am. J. Drug Alcohol Abus 35 (2009) 267–272, 10.1080/00952990903021638. 19591065

[37] Walter M, Bentz D, Schicktanz N, Milnik A, Aerni A, Gerhards C, Schwegler K, Vogel M, Blum J, Schmid O, Roozendaal B, Lang UE, Borgwardt S, de Quervain D, Effects of cortisol administration on craving in heroin addicts, Transl. Psychiatry 5 (2015), 10.1038/tp.2014.135. PMC5068724 26218852

[38] Kutscher S, Heise DJ, Banger M, Saller B, Michel MC, Gastpar M, Schedlowski M, Exton MS, Concomitant endocrine and immune alterations during alcohol intoxication and acute withdrawal in alcohol-dependent subjects, Neuropsychobiology 45 (2002) 144–149, 10.1159/000054913. 11979065

[39] Merry J, Marks V, The effect of alcohol, barbiturate, and diazepam on hypothalamic/pituitary/Adrenal Function in chronic alcoholics, Lancet 300 (1972) 990–992, 10.1016/S0140-6736(72)92686-5. 4116981

[40] Nava F, Caldiroli E, Premi S, Lucchini A, Relationship between plasma cortisol concentrations, withdrawal symptoms and craving in abstinent and treated heroin addicts, J. Addict. Dis 25 (2006) 9–16, 10.1300/J069v25n02_02. 16785214

[41] Risher-Flowers D, Adinoff B, Ravitz B, Bone GHA, Martin PR, Nutt D, Linnoila M, Circadian rhythms of cortisol during alcohol withdrawal, Adv. Alcohol Subst. Abus 7 (1) (1988) 37–41, 10.1300/J251V07N01_03. 3223434

[42] Adinoff B, Iranmanesh A, Veldhuis J, Fisher L, Disturbances of the stress response: the role of the HPA axis during alcohol withdrawal and abstinence, Alcohol Health Res. World 22 (1) (1998) 67–72, 10.1016/S0006-3223(98)00209-6. 15706736 PMC6761816

[43] Sinha R, The clinical neurobiology of drug craving, Curr. Opin. Neurobiol 23 (4) (2013) 649–654, 10.1016/j.conb.2013.05.001. 23764204 PMC3735834

[44] Nava F, Premi S, Manzato E, Campagnola W, Lucchini A, Gessa GL, Gamma-hydroxybutyrate reduces both withdrawal syndrome and hypercortisolism in severe abstinent alcoholics: An open study vs. Diazepam, Am. J. Drug Alcohol Abus 33 (2007) 379–392, 10.1080/00952990701315150. 17613965

[45] Piazza PV, Maccari S, Deminière JM Le Moal M, Mormède P, Simon H, Corticosterone levels determine individual vulnerability to amphetamine self-administration, Proc. Natl. Acad. Sci 88 (6) (1991) 2088–2092, 10.1073/pnas.88.6.2088. 2006148 PMC51174

[46] Huang M-C, Chen C-H, Chen L-Y, Chang H-M, Chen C-K, Lin S-K, Xu K, Chronic ketamine abuse is associated with orexin-a reduction and ACTH elevation, Psychopharmacology 237 (2019) 45–53, 10.1007/s00213-019-05269-5. 31377886

[47] Bannan LT, Potter JF, Beevers DG, Saunders JB, Walters JR, Ingram MC, Effect of alcohol withdrawal on blood pressure, plasma renin activity, aldosterone, cortisol and dopamine β-hydroxylase, Clin. Sci 66 (1984) 659–663, 10.1042/cs0660659. 6373096

[48] Meyrel M, Rolland B, Geoffroy PA, Alterations in circadian rhythms following alcohol use: A systematic review, Prog. Neuro-Psychopharmacol. Biol. Psychiatry 99 (2020) 109831, 10.1016/j.pnpbp.2019.109831. 31809833

[49] Motaghinejad M, Motaghinejad O, Motevalian M, Asadi-Ghalehni M, Attenuation of morphine withdrawal signs, blood cortisol and glucose level with forced exercise in comparison with clonidine, Adv. Biomed. Res 3 (2014) 171, 10.4103/2277-9175.137920. 25250285 PMC4166059

[50] Jukić T, Rojc B, Boben-Bardutzky D, Hafner M, Ihan A, The use of a food supplementation with D-phenylalanine, L-glutamine and L-5-hydroxytriptophan in the alleviation of alcohol withdrawal symptoms, Coll. Antropol 35 (2011) 1225–1230, 10.5671/ca.2011.35.4.11. 22397264

[51] Donoghue K, Rose A, Coulton S, Milward J, Reed K, Drummond C, Little H, Double-blind, 12 month follow-up, placebo-controlled trial of mifepristone on cognition in alcoholics: the MIFCOG trial protocol, BMC Psychiatry 16 (1) (2016) 61, 10.1186/s12888-016-0794-x. 26912003 PMC4765152

[52] Burov YV, Treskov VG, Vedernikova NN, Shevelyova OS, Types of alcohol withdrawal syndrome and dexamethasone suppression test, Drug Alcohol Depend. 17 (1986) 81–88, 10.1016/0376-8716(86)90010-1. 3720534

[53] Jeffcoate WJ, Silverstone JT, Edwards CRW, Besser GM, Psychiatric manifestations of Cushing’s syndrome: Response to lowering of plasma cortisol, QJM: Int. J. Med 48 (1979) 465–472, 10.1093/oxfordjournals.qjmed.a067091. 542586

[54] Khor B-S, Amar Jamil MF, Adenan MI, Chong Shu-Chien A, Mitragynine attenuates withdrawal syndrome in morphine-withdrawn zebrafish, PLoS ONE (2011) 6, 10.1371/journal.pone.0018298. PMC3244390 22205946

[55] Ravi SD, Dorus W, Park YN, Collins MC, Reid RW, Borge GF, The dexamethasone suppression test and depressive symptoms in early and late withdrawal from alcohol, Am. J. Psychiatry 141 (12) (1984) 1445–1448, 10.1176/ajp.141.12.1445. 6496789

[56] Wen D, Zhao P, Hui R, Wang J, Shen Q, Gong M, Guo H, Cong B, Ma C, Hydrogen-rich saline attenuates anxiety-like behaviors in morphine-withdrawn mice, Neuropharmacology 118 (2017) 199–208, 10.1016/j.neuropharm.2017.03.035. 28359771

[57] Moriarty AS, Bradley AJ, Anderson KN, Watson S, Gallagher P, McAllister-Williams RH, Cortisol awakening response and spatial working memory in man: A U-shaped relationship, Hum. Psychopharmacol.: Clin. Exp 29 (3) (2014) 295–298, 10.1002/hup.2398. 24911579

[58] Wirth MM, Hormones, stress, and cognition: The effects of glucocorticoids and oxytocin on memory, Adapt. Hum. Behav. Physiol 1 (2014) 177–201, 10.1007/s40750-014-0007-1. PMC4399826 25893159

[59] Wingenfeld K, Wolf OT, Effects of cortisol on cognition in major depressive disorder, posttraumatic stress disorder and borderline personality disorder - 2014 curt richter award winner, Psychoneuroendocrinology 51 (2015) 282–295, 10.1016/j.psyneuen.2014.10.025. 25462901

[60] Light LL, Singh A, Implicit and explicit memory in young and older adults, J. Exp. Psychol. Learn., Mem., Cogn 13 (4) (1987) 531–541, 10.1037//0278-7393.13.4.531. 2959737

[61] Olton DS, Spatial memory, Sci. Am 236 (1977) 82–98, 10.1038/scientificamerican0177-82. 867028

[62] Shields GS, Bonner JC, Moons WG, Does cortisol influence core executive functions? A meta-analysis of acute cortisol administration effects on working memory, inhibition, and set-shifting, Psychoneuroendocrinology 58 (2015) 91–103, 10.1016/j.psyneuen.2015.04. 25973565

[63] Vaz LJ, Pradella-Hallinan M, Bueno OF, Pompéia S, Acute glucocorticoid effects on the multicomponent model of working memory, Hum. Psychopharmacol.: Clin. Exp 26 (2011) 477–487, 10.1002/hup.1197. 21953602

[64] Beluche I, Carrière I, Ritchie K, Ancelin ML, A prospective study of diurnal cortisol and cognitive function in community-dwelling elderly people, Psychol. Med 40 (6) (2010) 1039–1049, 10.1017/S0033291709991103. 19814852 PMC2894868

[65] Echouffo-Tcheugui JB, Conner SC, Himali JJ, Maillard P, DeCarli CS, Beiser AS, Vasan RS, Seshadri S, Circulating cortisol and cognitive and structural brain measures: The Framingham Heart Study, Neurology 91 (21) (2018) e1961–e1970, 10.1212/WNL.0000000000006549. 30355700 PMC6260201

[66] Geerlings MI, Sigurdsson S, Eiriksdottir G, Garcia ME, Harris TB, Gudnason V, Launer LJ, Salivary cortisol, brain volumes, and cognition in community-dwelling elderly without dementia, Neurology 85 (11) (2015) 976–983, 10.1212/WNL.0000000000001931. 26291281 PMC4567466

[67] Holleman J, Adagunodo S, Kåreholt I, Hagman G, Aspö M, Udeh-Momoh CT, Solomon A, Kivipelto M, Sindi S, Cortisol, cognition and Alzheimer’s disease biomarkers among memory clinic patients, BMJ Neurol. Open 4 (2) (2022) e000344, 10.1136/bmjno-2022-000344. PMC9582323 36277478

[68] Ouanes S, Popp J, High cortisol and the risk of dementia and alzheimer’s disease: A review of the literature, Front. Aging Neurosci 11 (2019), 10.3389/fnagi.2019.00243. PMC6405479 30881301

[69] Cornelisse S, Joëls M, Smeets T, A randomized trial on mineralocorticoid receptor blockade in men: Effects on stress responses, selective attention, and memory, Neuropsychopharmacology 36 (13) (2011) 2720–2728, 10.1038/npp.2011.166. 21881569 PMC3230495

[70] Forget H, Lacroix A, Somma M, Cohen H, Cognitive decline in patients with Cushing’s syndrome, J. Int. Neuropsychol. Soc 6 (2000) 20–29, 10.1017/s1355617700611037. 10761364

[71] Maldonado EF, Fernandez FJ, Trianes MV, Wesnes K, Petrini O, Zangara A, Enguix A, Ambrosetti L, Cognitive performance and morning levels of salivary cortisol and α-amylase in children reporting high vs. low daily stress perception, Span. J. Psychol 11 (2008) 3–15, 10.1017/S1138741600004184. 18630643

[72] Seed JA, Dixon RA, McCluskey SE, Young AH, Basal activity of the hypothalamic-pituitary-adrenal axis and cognitive function in anorexia nervosa, Eur. Arch. Psychiatry Clin. Neurosci 250 (2000) 11–15, 10.1007/s004060070020. 10738859

[73] Vedhara K, Hyde J, Gilchrist ID, Tytherleigh M, Plummer S, Acute stress, memory, attention and cortisol, Psychoneuroendocrinology 25 (2000) 535–549, 10.1016/S0306-4530(00)00014-5. 10840167

[74] Köhler S, Thomas AJ, Lloyd A, Barber R, Almeida OP, O’Brien JT, White matter hyperintensities, cortisol concentrations, brain atrophy, and continuing cognitive deficits in late-life depression, Br. J. Psychiatry 196 (2010) 143–149, 10.1192/bjp.bp.109.067058. 20118461

[75] McLennan SN, Ihle A, Steudte-Schmiedgen S, Kirschbaum C, Kliegel M, Hair cortisol and cognitive performance in working age adults, Psychoneuroendocrinology 67 (2016) 100–103, 10.1016/j.psyneuen.2016.02.019. 26881835

[76] Keller J, Gomez R, Williams G, Lembke A, Lazzeroni L, Murphy GM, Schatzberg AF, HPA axis in major depression: Cortisol, clinical symptomatology and genetic variation predict cognition, Mol. Psychiatry 22 (2016) 527–536, 10.1038/mp.2016.9. 27528460 PMC5313380

[77] Havelka D, Prikrylova-Kucerova H, Prikryl R, Ceskova E, Cognitive impairment and cortisol concentrations in first-episode schizophrenia patients, Stress 19 (2016) 383–389, 10.1080/10253890.2016.1187271. 27320489

[78] Lenze EJ, Hershey T, Newcomer JW, Karp JF, Blumberger D, Anger J, Doré P, Dixon D, Antiglucocorticoid therapy for older adults with anxiety and co-occurring cognitive dysfunction: Results from a pilot study with mifepristone, Int. J. Geriatr. Psychiatry 29 (2014) 962–969, 10.1002/gps.4064. 24633761 PMC4138285

[79] Lupien SJ, Gaudreau S, Tchiteya BM, Maheu F, Sharma S, Nair NP, Hauger RL, McEwen BS, Meaney MJ, Stress-induced declarative memory impairment in healthy elderly subjects: Relationship to cortisol reactivity1, J. Clin. Endocrinol. Metab 82 (1997) 2070–2075, 10.1210/jcem.82.6.4055. 9215274

[80] Lupien SJ, King S, Meaney MJ, McEwen BS, Can poverty get under your skin? basal cortisol concentrations and cognitive function in children from low and high socioeconomic status, Dev. Psychopathol 13 (2001) 653–676, 10.1017/S0954579401003133. 11523853

[81] Malcolm R, “there’s no constant”: Oxytocin, cortisol, and balanced proportionality in hormonal models of autism, Med. Anthropol 40 (2021) 375–388, 10.1080/01459740.2021.1895071. 36018784

[82] Newcomer JW, Craft S, Askins K, Hershey T, Bardgett ME, Csernansky JG, Gagliardi AE, Vogler G, Glucocorticoid interactions with memory function in schizophrenia, Psychoneuroendocrinology 23 (1998) 65–72, 10.1016/S0306-4530(97)00083-5. 9618753

[83] Sampedro-Piquero P, Vicario S, Pérez-Rivas A, Venero C, Baliyan S, Santín L, Salivary cortisol concentrations are associated with craving and cognitive performance in cocaine-abstinent subjects: A pilot study, Brain Sci. 10 (2020) 682, 10.3390/brainsci10100682. 32992573 PMC7600918

[84] Keller J, Gomez R, Williams G, Lembke A, Lazzeroni L, Murphy GM Jr, Schatzberg AF, HPA axis in major depression: cortisol, clinical symptomatology and genetic variation predict cognition, Mol. Psychiatry 22 (4) (2017) 527–536, 10.1038/mp.2016.120. 27528460 PMC5313380

[85] Contalbrigo L, De Santis M, Toson M, Montanaro M, Farina L, Costa A, Nava F, The efficacy of dog assisted therapy in detained drug users: A pilot study in an Italian attenuated custody institute, Int. J. Environ. Res. Public Health 14 (2017) 683, 10.3390/ijerph14060683. 28672787 PMC5551121

[86] Drinkhouse M, Birmingham SSW, Fillman R, Jedlicka H, Correlation of human and horse heart rates during equine-assisted therapy sessions with at-risk youths: A pilot study, J. Stud. Res 1 (2012) 22–25, 10.3818/JSR.1.1.2012.22.

[87] Naber A, Kreuzer L, Zink R, Millesi E, Palme R, Hediger K, Glenk LM, Heart rate, heart rate variability and salivary cortisol as indicators of arousal and synchrony in clients with intellectual disability, horses and therapist during equine-assisted interventions, Pet. Behav. Sci (2019) 17–23, 10.21071/pbs.v5i1.11081.

[88] Pendry P, Carr AM, Vandagriff JL, Adolescents’ affective and physiological regulation shape negative behavior during challenging Equine Assisted Learning Activities, Front. Vet. Sci 5 (2018) 43, 10.3389/fvets.2018.00043. 30564583 PMC6288444

[89] Pendry P, Smith AN, & Roeter SM (n.d.). Randomized trial examines effects of equine facilitated learning on … Human-Animal Interaction Bulletin. Retrieved from 〈https://s3.wp.wsu.edu/uploads/sites/609/2014/04/effects-of-equine-facilitated-learning.pdf〉.

[90] Kang OD, Yun YM, Influence of Horse and Rider on Stress during Horse-riding Lesson Program, Asian-Australas. J. Anim. Sci 29 (6) (2016) 895–900, 10.5713/ajas.15.1068. 27004819 PMC4852258

[91] Munsters CCBM, Visser KEK, van den Broek J, Sloet van Oldruitenborgh-Oosterbaan MM, The influence of challenging objects and horse-rider matching on heart rate, heart rate variability and behavioural score in riding horses, Vet. J 192 (1) (2012) 75–80, 10.1016/j.tvjl.2011.05.021. 21612959

[92] Lanata A, Guidi A, Valenza G, Baragli P, & Scilingo EP (2017). The role of nonlinear coupling in human-horse interaction: A preliminary study. 2017 39th Annual International Conference of the IEEE Engineering in Medicine and Biology Society (EMBC). 10.1109/EMBC.2017.8037245. 29060119

[93] Bridgeman DJ (2009). The working relationship between horse and rider during training and competition for Equestrian Sports. USQ ePrints. 〈http://eprints.usq.edu.au/id/eprint/6533〉.

[94] Gehrke EK (2012). The Horsehuman Heart Connection. Blissful Existence Healing Acres. Retrieved from 〈https://blissfulexistencehealingacres.com/wp-content/uploads/2021/09/BEHA-Research_The-Horse-Human-Heart-Connection-1.pdf〉.

[95] Peeters M, Closson C, Beckers J, Vandenheede M, Rider and Horse Salivary Cortisol Levels During Competition and Impact on Performance, J. Equine Vet. Sci 33 (2013) 155–160, 10.1016/j.jevs.2012.05.073.

[96] Broadfield K, Can Animal-assisted Therapy Aid Recovery from Alcohol and Drug Addiction? A Brief Review of the Literature, Res. Gate 2 (2020).

[97] Campbell-Begg T. (1998). Promotion of transactions during animal assisted, group therapy with individuals who are recovering from chemical addictions [Master’s thesis, D′Youville College School of Health and Human Services]. ProQuest Dissertations and Theses database. (UMI No. 134236)

[98] Sikstrom L, Meyer T, Katz E, Choi MM, Darragh M, Cutler-Palma A, Conforti T, Kalocsai C, Soklaridis S, Increasing participation in research with therapy dogs: A qualitative study at a large urban mental health and addiction hospital, PloS One 15 (8) (2020) e0238096, 10.1371/journal.pone.0238096. 32853258 PMC7451510

[99] Wesley MC, Minatrea NB, Watson JC, Animal-assisted therapy in the treatment of substance dependence, AnthrozoöS. 22 (2009) 137–148, 10.2752/175303709X429163.

[100] Kern-Godal A, Brenna IH, Arnevik EA, Ravndal E, More than just a break from treatment: How substance use disorder patients experience the stable environment in horse-assisted therapy, Subst. Abus.: Res. Treat (2016) 10, 10.4137/SART.S38149. PMC5054942 27746677

[101] Bark J. (2011). Therapists working together with Horses Equine Assisted Psychotherapy: Treating youths with addiction. Diva Portal. 〈https://www.diva-portal.org/smash/get/diva2:499300/FULLTEXT01.pdf〉.

[102] Contalbrigo L, Borgi M, De Santis M, Collacchi B, Tuozzi A, Toson M, Redaelli V, Odore R, Vercelli C, Stefani A, Luzi F, Valle E, Cirulli F, Equine-assisted interventions (eais) for children with autism spectrum disorders (ASD): Behavioural and physiological indices of stress in domestic horses (Equus caballus) during riding sessions, Animals 11 (2021) 1562, 10.3390/ani11061562. 34071859 PMC8227027

[103] Gelvin-Smith C. (2017). Equine assisted therapy: Supporting treatment for Substance Use Disorders in Alaska. Handle Proxy. Retrieved from 〈http://hdl.handle.net/11122/7958〉.

[104] Kern-Godal A, Brenna IH, Kogstad N, Arnevik EA, Ravndal E, Contribution of the patient-horse relationship to substance use disorder treatment: Patients’ experiences, Int. J. Qual. Stud. Health Well-being 11 (2016) 31636, 10.3402/qhw.v11.31636. 27291162 PMC4904069

[105] Scott TM, Kirnan J, When are the dogs coming back? animal-assisted activities with men in residential substance abuse treatment, Humanist. Psychol. Adv. Online Publ (2021), 10.1080/08873267.2021.1963244.

[106] Uhlmann C, Nauss C, Worbs A, Pfund U, Schmid P, Effekte einer tiergestützten intervention in der Stationären psychiatrischen suchtbehandlung – Eine Pilotstudie [Effects of animal-assisted intervention in stationary psychiatric addiction treatment - A pilot study], Fortschr. der Neurol. ⋅ Psychiatr 87 (2019) 305–311, 10.1055/s-0043-118239. 30791060

[107] Adams C, Arratoon C, Boucher J, Cartier G, Chalmers D, Dell CA, Dell D, Dryka D, Duncan R, Dunn K, Hopkins C, Longclaws L, MacKinnon T, Sauve E, Spence S, Wuttunee M, The helping horse: how equine assisted learning contributes to the wellbeing of first nations youth in treatment for volatile substance misuse, Hum. -Anim. Interact. Bull 3 (1) (2015) 52–75, 10.1037/h0100782. PMC4716821 26793794

[108] Dell C, Questioning “fluffy”: A dog’s eye view of animal-assisted interventions (AAI) in the treatment of Substance Misuse, Subst. Use Misuse 50 (3) (2015) 1148–1152, 10.3109/10826084.2015.1007657. 25775152 PMC4636384

[109] Dell CA, Chalmers D, Dell D, Suave E, MacKinnon T, Horse as Healer: An Examination of Equine Assisted Learning in the Healing of First Nations Youth from Solvent Abuse, A J. Aborig. Indig. Community Health 6 (1) (2008) 40–56, 10.18357/ijih61200812310.

[110] Dell CA, Chalmers D, Bresette N, Swain S, Rankin D, Hopkins C, A healing space: The experiences of First Nations and Inuit youth with equine-assisted learning (EAL), Child Youth Care Forum 40 (4) (2011) 319–336, 10.1007/s10566-011-9147-6.

[111] Frewin K, David B, New Age or Old Sage? A review of equine-assisted psychotherapy, Aust. J. Couns. Psychol 6 (2005) 13–17, 10.1375/acp.6.3.13.

[112] Kelly MA, Cozzolino CA, Helping at-risk youth overcome trauma and substance abuse through animal-assisted therapy, Contemp. Justice Rev 18 (2015) 421–434, 10.1080/10282580.2015.1075908.

[113] Coetzee N, Beukes JT, Lynch I, Substance abuse inpatients’ experience of animal-assisted therapy, J. Psychol. Afr 23 (2013) 477–480, 10.1080/14330237.2013.10820506.

[114] Husband A, Ahmed A, Dell CA, An exploratory case study of the impact of psychiatric service dogs on problematic substance use among PTSD-diagnosed veterans, J. Subst. Use 25 (2019) 113–117, 10.1080/14659891.2019.1571599.

[115] Monfort M, Benito A, Haro G, Fuertes-Saiz A, Cañabate M, Baquero A, The efficacy of animal-assisted therapy in patients with dual diagnosis: Schizophrenia and addiction, Int. J. Environ. Res. Public Health 19 (12) (2022) 6695, 10.3390/ijerph19126695. 35682281 PMC9180053

[116] Birkmayer F, Equine-assisted psychotherapy for substance use and co-occurring disorders, in: Modir S, Munoz G. (Eds.), Integrative Addiction and Recovery, Oxford University Press, 2018, pp. 423–426.

[117] Gatti F, Walderhaug E, Kern-Godal A, Lysell J, Arnevik EA, Complementary horse-assisted therapy for Substance Use Disorders: A randomized controlled trial, Addict. Sci. Clin. Pract (2020) 15, 10.1186/s13722-020-00203-5. PMC7001193 32019584

